# Osteoinductive and Osteogenic Capacity of Freeze-Dried Bovine Bone Compared to Deproteinized Bovine Bone Mineral Scaffold in Human Umbilical Cord Mesenchymal Stem Cell Culture: An *In Vitro* Study

**DOI:** 10.1055/s-0042-1758786

**Published:** 2023-01-04

**Authors:** Andreas Pratama Nugraha, David B. Kamadjaja, Ni Putu Mira Sumarta, Andra Rizqiawan, Coen Pramono, Anita Yuliati, Eryk Hendrianto, Mohammad Zeshaan Rahman

**Affiliations:** 1 Magister Program of Clinical Medicine, Faculty of Medicine, Universitas Airlangga, Surabaya, Indonesia; 2 Department of Oral and Maxillofacial Surgery, Faculty of Dental Medicine, Universitas Airlangga, Surabaya, Indonesia; 3 Departement of Dental Material, Faculty of Dental medicine Universitas Airlangga, Surabaya, Indonesia; 4 Stem Cell Research and Developmental Center, Universitas Airlangga, Surabaya, Indonesia; 5 Department of Oral and Maxillofacial Surgery, Pioneer Dental College and Hospital, Dhaka, Bangladesh

**Keywords:** runt-related transcription factor-2, osterix, human umbilical mesenchymal stem cells, osteoinductive, medicine

## Abstract

**Objective** Freeze-dried bovine bone scaffold (FDBB) or decellularized FDBB (dc-FDBB) was developed as an ideal scaffold with osteoinductive properties. This research aims to compare the osteoinductive properties marked by the expression of runt-related transcription factor-2 (RUNX2) and Osterix (OSX) and the osteogenic capacity of these scaffolds imbued with human umbilical cord mesenchymal stem cells (hUCMSCs).

**Materials and Methods** This study was performed in five experimental groups: a negative control group (C-) of hUCMSCs with a normal growth medium, a positive control group (C + ) of hUCMSCs with an osteogenic medium, experimental group 1 (E1) with an FDBB conditioned medium (CM), and experimental group 2 (E2) with a dc-FDBB-CM, and a third experimental group (E3) consisting of a DBBM-CM. Alizarin red staining was performed to qualitatively assess osteoinductive capacity. RUNX2 and OSX expression was quantified using real-time quantification polymerase chain reaction with two replications on day six (D6) and day 12 (D12) as fold changes.

**Results** This experiment revealed that hUCMSCs were positively expressed by CD73, CD90, and CD105 but were not expressed by CD34. Alizarin red staining showed that E1 had the most calcium deposition on D6 and D12, followed by E3 and then E2 The RUNX2 and OSX expression was higher in E1 but this difference was not significant. The OSX expression in E1,E2,E3 was lower on D12 and C+ of OSX had the highest expression. There was a significant difference of fold change measured between all groups ( *p* < 0.05), and there was no significant difference between any of the groups treated with OSX and RUNX2 on D6 and D12.

**Conclusion** FDBB osteoinduction and osteogenic capacity were higher when compared with DBBM and dc-FDBB.

## Introduction

Maxillofacial bone defects arise from maxillofacial trauma, periodontal disease, surgical excision, infection or congenital malformations, and oral neoplasia or cancer, and its reconstruction remains a common challenge in the daily practice of oral and maxillofacial surgery because it affects stomatognathic function and facial aesthetics. 1 The use of bone graft material and its substitutes in maxillofacial surgery for bony reconstruction has significantly increased in recent years. Current global statistics indicate that ∼2.2 million bone grafting procedures are being performed every year, and the number of procedures to reconstruct bony defects is estimated to increase by 13% in the next few years. 2 3

Reconstruction methods using xenograft, mainly derived from deproteinized bovine bone which have been widely developed and applied. Although autograft is considered to be the gold standard because it often result in donor site morbidity. 2 Currently, the major problem with bovine bone xenografts commonly used in regenerative surgeries is that these materials, despite their good mechanical stability, are mainly osteoconductive scaffolds with slow degradation 4 8 Investigation into the development of bovine bone scaffolds that retain their organic components is essential for tissue engineering, as they are potentially osteoinductive, owing to their biologically active growth factors. However, they have good mechanical stability and are completely biodegradable, with an ideal resorption time when combined with the cell osteogenic properties 8 of the host.

The main disadvantage of xenografting is immunorejection, often referred to as delayed xenograft rejection, which destroys graft less than 2 week. This condition is now considered to be a major immunologic barrier to successful xenotransplantation. 5 The material processing method for xenografting was developed with freeze drying or lyophilization, resulting in freeze-dried bovine bone (FDBB), decellularized FDBB (dc-FDBB), demineralized FDBB (DFDBB), and deproteinized bovine bone mineral (DBBM). 6 DFDBB has good osteoinductive capacity but lacks osteoconductive capacity because the demineralization process compromises its biomechanical properties and enhances graft resorption. DBBM has good osteoconductive and biomechanical properties but lacks osteoinductive capacity because the deproteinization process destroys organic material and growth factors within. 7 FDBB has become a candidate for an ideal scaffold because freeze drying preserves the protein and growth factor without compromising biomechanical properties. 8 Decellularization of FDBB aims to decrease the risk of immunorejection and create a natural microenvironment but often results in a decrease in the osteoinductive capacity. 9

Runt-related transcription factor 2 (RUNX2) and osterix (OSX) play a pivotal role in determining osteoinductivity and are key transcription factors in the early phase of osteogenesis that regulate osteoprogenitor cell differentiation into pre-osteoblasts. 10 11 OSX is a downstream gene of RUNX2, which is essential to inducing differentiation of pre-osteoblasts into mature and functional osteoblasts. 12 RUNX2 has been identified as “a master gene” for the differentiation of osteoblasts, with a pivotal role in both the intramembranous and endochondral ossification processes of osteogenesis. 10 RUNX *2* -deficient mice have demonstrated a complete absence of cartilage and mature osteoblasts and ossification with only fibrous tissue evident. OSX deficiency in knockout mice shows normal cartilage formation without mineralization, further resulting in delayed calvarias ossification and callus fractures at multiple skeletal sites. 13 FDBB contains growth factors, especially bone morphogenetic protein-2 (BMP-2), which activates the BMP/SMAD pathway and canonical wnt/β catenin pathway to upregulate the RUNX2 and OSX synthesis on the cell nucleus. 8 13 The osteoinductive and osteodifferentiation capacity of FDBB are still unclear and require further study. Furthermore, this study aims to investigate the osteoinductive and osteodifferentiation capacity of an FDBB scaffold on human umbilical cord mesenchymal stem cells (hUCMSCs). This research hypothesizes that the expression of *RUNX2* and *OSX* genes is higher in FDBB than in dc-FDBB and DBBM on day 6 (D6) and day 12 (D12).

## Materials and Methods

### Study Design and Ethical Clearance

This study has a true experimental in vitro post-test-only control group design. An ethical clearance certificate of approval for the experimental study was issued by the Faculty of Dental Medicine, Universitas Airlangga, Surabaya, Indonesia, with a reference number of 334/HRECC.FODM/VI/2021. The research was performed at the Universitas Airlangga Stem Cell Research and Developmental Center Laboratory.

### Isolation and Culture of Human Umbilical Cord Mesenchymal Stem Cells

A cesarean section was performed in the Central Operating Theater of the Dr. Soetomo General Hospital, Surabaya, on a healthy full-term neonate. Consent was obtained from the patient's guardian and the necessary forms were completed. Isolation and culture of the hUCMSCs were done according to the method developed by Hendrijatini. 15 The umbilical cord was dissected into 1 cm pieces, and the umbilical arteries, veins, and adventitia were removed to obtain Wharton's jelly. Wharton's jelly was minced with a knife into 1 to 3 mm pieces and was used to isolate and culture primary hUCMSCs. Wharton's jelly was transferred to 0.25% trypsin and digested at 37°C for 40 minutes. It was then centrifuged and the supernatant was removed. This process was repeated twice. The crushed and digested sample was then subjected to phosphate-buffered saline (PBS) containing 0.75 mg/mL collagenase IV (Sigma-Aldrich, St. Louis, MO, USA) and 0.075 mg/mL DNase I (Takara Bio, Shiga, Japan) and incubated at 37°C for 60 minutes. It was then filtered using a cell strainer and pellet collection upon centrifugation for 10 minutes to obtain the final cells. The single cells collected were then cultured in collagen-coated dishes using α modification of minimum essential medium (α-MEM; Gibco BRL Accessories, Gaithersburg, MD, USA) supplemented with human leukemia inhibitory factor (10 ng/mL) and fetal bovine serum (FBS; Gibco BRL Accessories). Primary cell growth was observed under a microscope, the timing of cell confluence was recorded, and the medium was changed once every 3 days. After the confluence reached 80%, cell splitting was done using trypsin. One-half to two-thirds of the cells were then replated onto a new dish of the same medium. 14

### Human Umbilical Cord Mesenchymal Stem Cell Characterization

Characteristics of the harvested cells were confirmed with immunofluorescence by applying clusters of differentiation (CD) of CD34, CD 73, CD 90, and CD 105 as markers, according to the method suggested by Hendrijatini, 15 following the experimental protocol as follows: hUCMSCs were washed three times with PBS and then with a 1:100 dilution of primary antibodies from CD 45, CD 73, CD 90, and CD 105 (Santa Cruz Biotechnology, Santa Cruz, CA, USA), which were added with fluorescein isothiocyanate (FITC)-conjugated antibodies. In addition, a total of 5 × 10 ^5^ cells were resuspended in 0.2 mL Phosphate Buffer Saline (PBS) and incubated with 10 µL of FITC-conjugated antibodies for 30 minutes at room temperature. The fluorescence intensity of the cells was evaluated using FACScan (BD Biosciences, New Jersey, USA), and the data were then analyzed using CellQuest software version 6.0 (BD Biosciences, United Kingdom). 14

### Alizarin Red Staining

Alizarin red staining was done using a Cyagen kit (MoBiTec Molecular Biotechnology, Goettingen, Germany), using the following procedure: the hUCMSCs were osteogenically differentiated and stained with alizarin red staining. After the cells were differentiated, the osteogenic differentiation medium was removed from the wells and rinsed with 1× PBS. The cells were fixed with 2 mL of 4% formaldehyde solution for 30 minutes. The wells were rinsed twice with 1× PBS and then stained with 1 mL of alizarin red staining working solution for 3 to 5 minutes. The wells were rinsed two to three times with 1× PBS. The cells could then be visualized and analyzed under a microscope at 40× magnification (CX22 Binocular, Olympus, Tokyo, Japan).

### Scaffold-conditioned Medium and human umbilical cord mesenchymal stem cell exposure

A scaffold-conditioned medium (CM) was made using a protocol from previous research by Filho. 16 FDBB, dc-FDBB, and the DBBM block scaffold sized 10 × 5 × 5 mm were obtained from the Tissue Bank at the Dr. Soetomo General Academic Hospital, Surabaya, with a production date of December 14, 2021. Each was immersed in α-MEM, antibiotic penicillin (100 units), and L-glutamine (2 mM) for 2 days. The ratio of scaffold to growth medium was 1 g of scaffold to 10 mL of culture medium (10% weight/volume). After 2 days of immersion, centrifugation was done at 600 g force for 8 minutes at 20°C. The supernatant was then filtered using a 0.22 mM filter, resulting in FDBB, dc-FDBB, and DBBM CMs.

The hUCMSC exposure to the scaffold CM: normal growth medium was done at the optimal ratio of 3:7, based on the results of previous research methods 16 for modulating the expression of the osteodifferentiation process. The sterile scaffold CM was placed on two sets of M6 plates, consisting of six wells, each with a volume of 2 mL and observed on two days (D6 and D12). The first well consisted of an FDBB–CM, the second well consisted of dc-FDBB-CM, the third well consisted of DBBM-CM, the fourth well consisted of the osteogenic medium (MesenCult Osteogenic Differentiation Kit–(STEMCELL Technologies, Vancouver, Canada), and the fifth well consisted of Alpha MEM. hUCMSCs were suspended in each well with a ratio of 10 ^6^ cells in 200 µL of the medium. The suspension was first given 100 µL using an in-out pipette and then incubated for 60 minutes at 37°C, 98% humidity, and 5% CO _2_ concentration. The second suspension was given 100 µL and incubated under the same conditions as described above. The seeded well was moved to the sterile well with a new culture medium consisting of α-MEM, 10% FBS, 100 units of penicillin, and 2 mM L-glutamine. The culture medium was replaced every 3 days.

### Examination of Runt-Related Transcription Factor-2 and Osterix expression using Reverse Transcription Polymerase Chain Reaction

RUNX2 and OSX expression were examined using the automatic reverse transcription polymerase chain reaction (RT-PCR) machine (Applied Biosystem 7500 Fast Thermo Fisher, Massachussetts), RNA extraction kit (TIANGEN, Beijing, China), GoScript (Promega, Madison, Wincosin, USA), GoTaq qPCR master mix (Promega, Madison, Wincosin, USA). The primer was designed using the National Center Biological Information–Basic Local Alignment Search Tools Web site with specific criteria and was confirmed using Primer3Plus on the Web site until the primer sequence was acceptable: primer sequence RUNX2 forward 5′-ATTCGCCTCACAAACAACCA-3′ and reverse 5′-CTGCTTGCAGCCTTAAATGAC-3′, primer sequence OSX forward 5′-GGGATGGAGGCGAGATCC -3′ and reverse 5′-TCCACTCCTGTTCCACTCCAG -3′, and the internal control gene or housekeeping gene glyceraldehyde 3-phosphate dehydrogenase (GAPDH) forward 5′-CTCCTCCTGTTCGACAGTCA-3′ and reverse 5′-TGAGGTCAATGAAGGGGTCA -3′, with two sample replications for each gene.

The total ribonucleic acid (RNA) was collected from the cultures as well as from undifferentiated hUCMSCs with different passages. The TIANGEN reagent (Beijing, China) was used following the manufacturer's instructions. Briefly, cell layers were washed with PBS, scrapped, and homogenized in 1 mL TIANGEN. Later, RNA was treated with DNase I (Invitrogen, Waltham, Massachussets, USA) to remove genomic deoxyribonucleic acid (DNA), and 3 µg of total RNA was reverse transcribed to complementary (cDNA) using the iScript cDNA Synthesis Kit, 25R (Bio Rad Laboratories, Berkeley, California, USA) for RT-PCR (AB 7500 Fast) in conformity with the manufacturer's instructions. Furthermore, reverse transcription was performed in a thermomixer (Eppendorf, Hamburg, Germany) under the following conditions: 10 minutes at 20°C, 1 hour at 42°C, 5 minutes at 99°C and 5 minutes at 5°C. Gene expression was then determined by quantitative RT-PCR using GoTaq qPCR Master Mix (Promega, Madison, Wincosin, USA). Meanwhile, the primers used to determine gene expression was presented, and the PCR reaction conditions were as follows: 10 minutes at 95°C for one cycle, and 15 seconds at 95°C and 1 minute at 60°C for 40 cycles. Quantitative RT-PCR was performed on the 7500 Fast PCR system. Expression was normalized to β-actin. The relative expression of the targeted gene's fold change was calculated using the ΔΔCt method. 43 The first ΔCt was measured using Gene Ct (OSX and RUNX2) minus the internal control gene (GAPDH), then ΔΔCt was measured using ΔCt minus the average ct. The final fold change of each gene was measured using 2 ^-ΔΔCt^ .

### Statistical Analysis

SPSS version 20.0 for Windows (IBM Corporation, Illinois, Chicago, USA) was used to analyze the data. Data are described as mean and standard deviation. The distribution of data normality was examined using the Kolmogorov–Smirnov test, and homogeneity was tested using Levene's test. All data in each group were analyzed by employing analysis of variance to investigate the significance of the difference between groups, followed by a posthoc Tukey honest significant difference test (HSD). *p* < 0.05 was considered statistically significant.

## Results

**Fig. 1:**
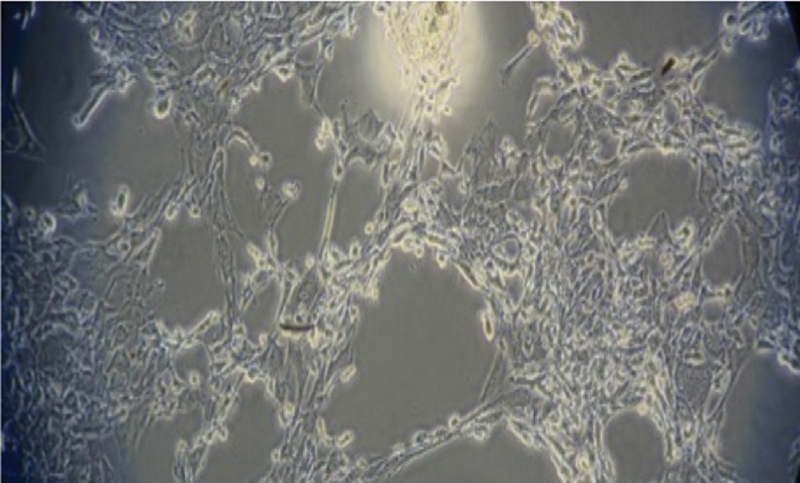
The hUCMSC morphology exhibited a spindle cell-like fibroblast and adhered to the culture plate's base. The examination was conducted using an electron microscope (CX22 Binocular, Olympus) at 200× magnification.

**Fig. 2:**
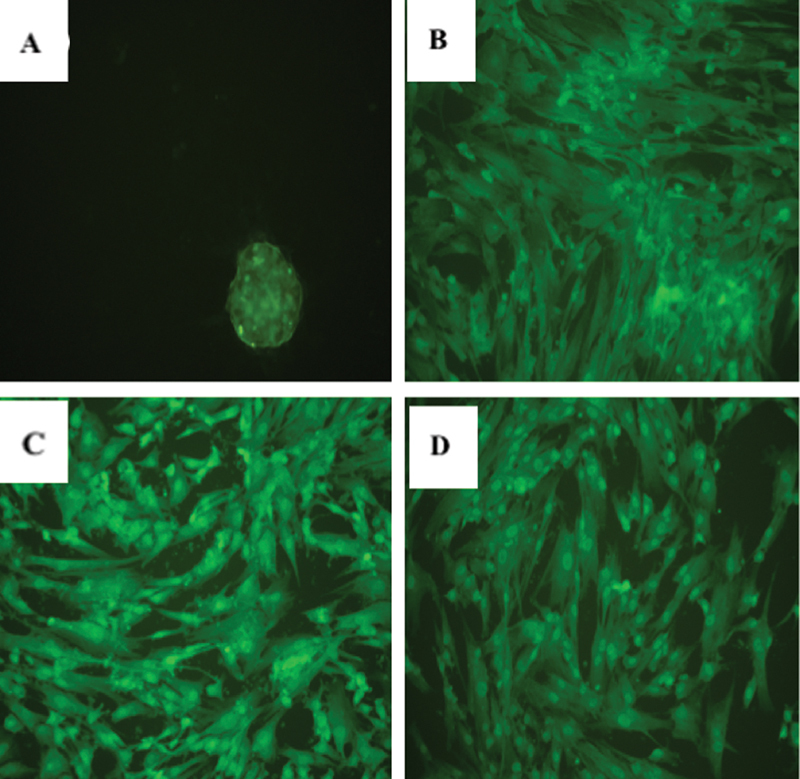
hUCMSC characterization using immunofluorescence. ( **A** ) negative for CD 34, ( **B** ) Positive for CD 73, ( **C** ) CD 90, and ( **D** ) CD 105. The examination was conducted using an electron microscope (CX22 Binocular, Olympus) at 100× magnification.

The hUCMSCs were successfully isolated from the umbilical cord. The spindle shape or fibroblast-like morphology was shown at the fifth subcultured hUCMSCs and had attached to the base of the culture plate ( Fig. 1 ). The characterization of hUCMSCs at the fifth subculture tested positive for the expression of CD 73, CD 90, and CD 105 but negative for the expression of CD 34 as MSCs surface markers ( Fig. 2 ).

Osteoinductivity capacity tests of scaffolds in hUCMSCs cultures were assessed by evaluating the expression of RUNX2 and OSX genes in cells through RT-PCR. First, the total RNA of the cells was isolated and converted into cDNA using a reverse transcriptase enzyme, and PCR was then performed on the cDNA. The expression of GAPDH was recorded as the internal control gene. The expression of RUNX2 and OSX in all treatment and control groups showed significant differences ( *p* < 0.05; Tables 1 and 2 ).

**Table 1:** RUNX2 expression analysis

Research group	Day 6 SD ± mean	Day 12 SD ± mean	*p-* Value
FDBB	0.55 ± 0.12	2.43 ± 0.15	*0.001
dc-FDBB	0.36 ± 0.22	1.74 ± 0.13	*0.001
DBBM	0.41 ± 0.05	2.36 ± 0.10	*0.001
Osteogenic medium	0.75 ± 0.07	0.21 ± 0.04	*0.001
α-MEM	0.14 ± 0.01	0.45 ± 0.17	*0.001

*information: *p-* Value < 0.05 it was a significant difference.

**Table 2:** OSX expression analysis

Research group	Day 6 SD ± mean	Day 12 SD ± mean	*p-* Value
FDBB	0.36 ± 0.46	0.25 ± 0.12	*0.01
dc-FDBB	0.28 ± 0.15	0.16 ± 0.27	*0.01
DBBM	0.57 ± 0.23	0.20 ± 0.02	*0.01
Osteogenic medium	0.18 ± 0.20	1.16 ± 0.15	*0.01
α-MEM	0.14 ± 0.16	0.17 ± 0.06	*0.01

*information: *p-* Value < 0.05 it was a significant difference.

**Fig. 3:**
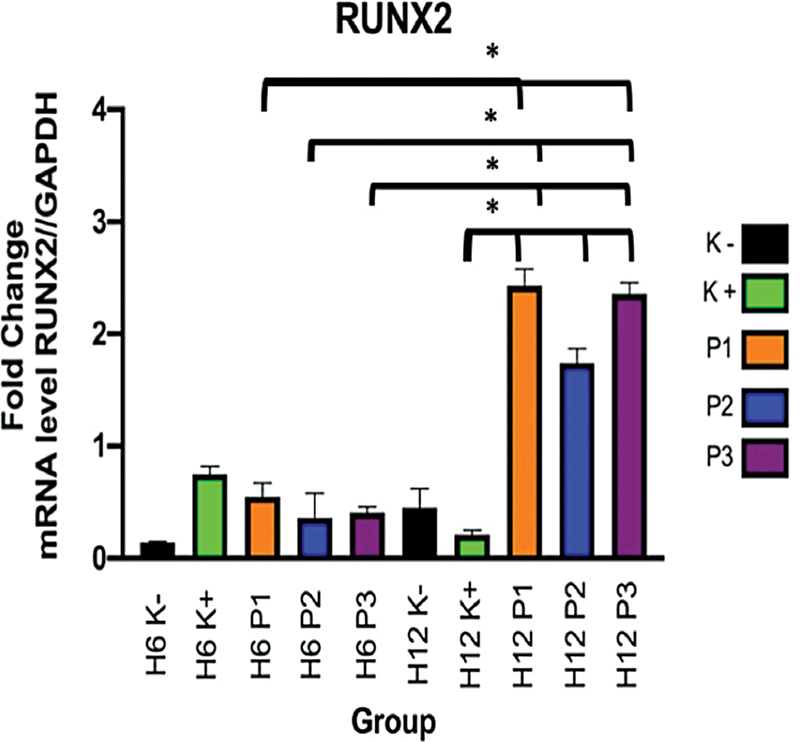
RT-PCR results for RUNX2. Information: a significant difference was found using the posthoc Tukey HSD ( *p* < 0.05). Color explanation: black is α-MEM medium-negative control (C − ), green is osteogenic medium positive control (C + ), orange is FDBB (E1), blue is dc-FDBB (E2), and purple is DBBM (E3). Examination days were D6 and D12.

The expression of RUNX2 in the osteogenic medium was the highest on D6 and then decreased by D12. In contrast, the expression of RUNX2 in the growth medium increased from D6 to D12. The expression of RUNX2 in FDBB significantly increased from D6 to D12 ( *p* < 0.05). The expression of RUNX2 in FDBB, dc-FDBB, and DBBM was significantly higher on D6 and D12 when compared with the osteogenic medium and the α-MEM medium ( *p* < 0.05; Fig. 3 **)** .

**Fig. 4:**
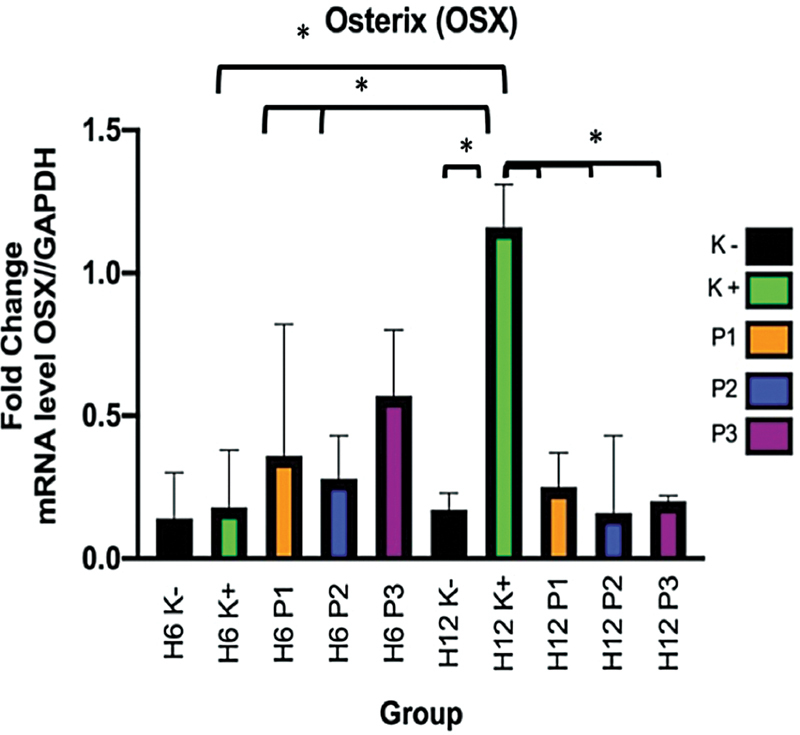
RT-PCR results for OSX. Information: a significant difference was found using the posthoc Tukey HSD ( *p* < 0.05). Color explanation: black is α-MEM medium negative control (C − ), green is osteogenic medium positive control (C + ), orange is FDBB (E1), blue is dc-FDBB (E2), and purple is DBBM (E3). Examination days were D6 and D12.

OSX expression upregulated significantly in the osteogenic medium between D6 and D12 ( *p* < 0.05). The expression of OSX in DBBM, dc-FDBB, and DBBM was lower on D12 than on D6, without significant differences between treatment groups ( Fig. 4 ).

**Fig. 5:**
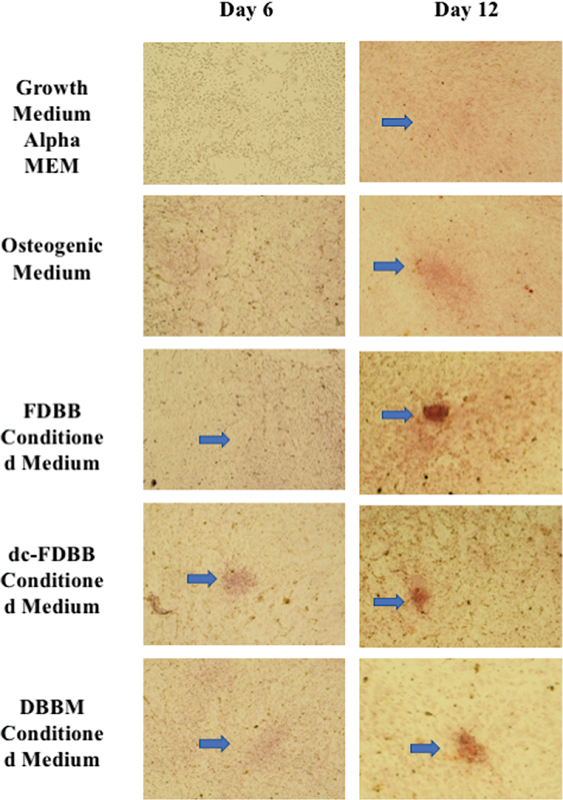
The alizarin red staining was examined using the electron microscope (CX22 Binocular, Olympus) at 40× magnification. The blue arrow indicates the focus area of calcification ( **A and B** ), The α-MEM medium showed no calcification. ( **C and D** ). The osteogenic medium, as the positive control, showed scarce calcification on D12. ( **E and F** ). The FDBB-CM showed a dense area of calcification on D12 **(G and H)** . The dc-FDBB-CM showed scarce calcification ( **I and J** ). The DBBM-CM showed quite dense area calcification.

Alizarin red staining was used in this research to qualitatively assess the formation of the mineralized matrix on the scaffolds. The results indicated the absorption of alizarin red dye on each scaffold CM, normal growth medium, and osteogenic medium due to calcium deposition by osteogenic differentiated cells. The intensity of absorbed dye on D6 was observed in the dc-FDBB, FDBB, and minimally in DBBM. The dye intensity was most clearly observed in a focused area in the FDBB, followed by the dc-FDBB and DBBM on D12, indicating the progress of osteogenic differentiation of cells during this period ( Fig. 5 ).

## Discussion

This study aimed to investigate the osteoinductive capacity and osteodifferentiation capacity of the FDBB scaffold. The tissue engineering scaffold principle was used to emphasize the well-known osteoconductive capacity of the scaffold into potential osteoinductive capacity when exposed to mesenchymal stem cells 18 . The FDBB is known for its osteoinductive and osteodifferentiation potential when compared with DBBM because it has organic components, such as collagen, non-collagen fiber, and growth factors, such as transforming growth factor β (TGFβ) and BMP-2 19 .

This study was an in vitro study using hUCMSCs. It used a multipotent cell that could differentiate into osteoblastic cell lineage, had osteogenic differentiation capacity, and did not easily become senescent. 20 One of the main problems in stem cell research is the uncontrolled differentiation of mesenchymal stem cells and progenitor cells. 21 To overcome this problem, we used a CM, which is known for its ability to induce osteoblastogenesis to minimize contamination from other cell lines. 22

The CM ratio was calculated as 3:7 between the scaffold CM and the basic growth medium, based on the results of previous research by Zhong, 17 which showed the remarkable ability of scaffold CM to upregulate the expression of osteoinductive cells, such as RUNX2, alkaline phosphatase, and osteocalcin. 17 Osteogenic gene profile examination is a very important process to validate the osteogenesis process. 23 RUNX2 and OSX were quantitatively measured on the messenger RNA levels using RT-PCR. 10

This study found that the highest RUNX2 expression was observed on D6 in the osteogenic medium group and on D12 in the FDBB group, which supports the results of a previous study that showed that the osteogenic medium consisted of ascorbic acid and β glycerol phosphate and that dexamethasone has the highest osteogenic gene expression because it is a potent and clinically proven osteoinducer. 24 The osteogenic medium has a similar osteoinductive capacity with its growth factor in modulating osteogenesis. 22 24 The expression of RUNX2 in FDBB significantly increased from D6 to D12, but no significant difference was found between the expression of RUNX2 in FDBB, dc-FDBB, and DBBM. The upregulation of RUNX2 observed on D12 supports the results from previous research by Duan, 25 which showed significant upregulation from D6 to D12. 25 RUNX2 expression initially appeared on the first day, increased by day 7, and reached its peak between days 14 and 21. It then significantly increased by 3 to 10-fold after a further 2 weeks of observation. 25 The results of the present study also concur with research by Chen et al, who stated that RUNX increased significantly on the seventh day and reached its peak between day 21 and day 28. Research by Furuya 27 and Marupanthorn 28 showed upregulation of RUNX2 on the third day, and stability on day 10, with the expression of RUNX2 reaching its peak on day 21. 27 hUCMSCs supplemented with BMP-2 exhibited superior RUNX2 expression when compared with the control group. 29 RUNX2 plays a vital role in the early phase of hUCMSC differentiation into pre-osteoblasts by upregulating specific osteoblastic differentiation by inducing another osteogenic marker such as Sp7 and the extracellular matrix (ECM). 13 Furthermore, a defect in RUNX2 expression will impact osteoblast maturation. 21

The results relating to OSX expression in this research showed no significant difference between FDBB, dc-FDBB, and DBBM on D6 or D12, but the expression of OSX decreased between D6 and D12. These results contradict the results of research by Hagh, 26 who stated that OSX expression significantly upregulated on the seventh day, reached its peak on day 14, with a 3 to 10-fold increase, and then decreased significantly by day 21. 26 OSX is an early marker of osteogenesis with an almost similar expression pattern as RUNX2. 27 Liu 30 stated that after 14 days of observation, both RUNX2 and OSX expression increased two to five-fold. 30 In the present study, OSX expression was the highest in the osteogenic medium group, which was significantly higher on D12 than on the other days, when compared with all control and treatment groups. Mukherje 31 explained that the OSX expression of an osteogenic culture medium with or without BMP supplementation appeared on the fifth and seventh days. 31 However, Valenti 32 stated that it appeared on the third day when mesenchymal stem cells were cultured with the osteogenic medium without BMP. 32 Chen 22 showed that OSX expression peaked on day 14 and then decreased after day 21. The present research showed that OSX expression decreased on D12 in the treatment group. 23 The explanation for this phenomenon could be that OSX has negative feedback capabilities in osteogenesis to avoid overexpression and ectopic bone formation by inhibiting osteoblastogenesis pathways, such as the Wnt/β catenin signaling pathway. 33 The biomolecular substances that could initiate the downregulation of OSX are long non-coding RNA (LncRNA) and osteogenic differentiation inhibitory regulator (ODIR1), which exclusively affect OSX. Both were downregulated by altered histone on the OSX promoter. Other research has shown that when LncRNA and ODIR1 were eliminated on the hUCMSCs, H2BK120 monoubiquitylation increased, which further stimulated the trimethylation of H3K4, significantly upregulating OSX expression. 34 The osteogenic medium group in this research showed the highest expression of OSX because it is considered a potent osteogenic inducer. 24 Another possibility is that the CM used in this study was made by mixing a basic growth medium and scaffolds in a 3:7 ratio. Thus, its osteoinductive capacity is still debatable, and further research into the optimal ratio is necessary. OSX is an important marker of osteogenesis because its function is to confirm that the osteoprogenitor specifically differentiates into the mature osteoblast and the upregulation of OSX is directly proportional to the bone regeneration capacity. 30

FDBB in this research showed remarkable osteoinductive capacity and superior calcium deposits in the most visible and focused areas of calcification. An explanation for this could be that the freeze-drying method preserved the organic components inside the bovine bone, such as ECM, collagen fiber, and glycosaminoglycans, which facilitated the medium in cell adhesion and proliferation. Organic components found in FDBB, such as BMP-2 and TGFβ, could induce osteogenetic signaling pathways, such as Wnt/β catenin and the BMP/SMAD pathway. 35 The considerable weakness of FDBB was due to lyophilization, which weakens structural stability and further shortens degradation time, resulting in graft resorption before optimum bone regeneration is achieved. 36

dc-FDBB was developed using decellularization method to eliminate its immunogenic component, which often resulting in delayed xenograft rejection. Elimination of these substances and the preservation of ECM to provide a natural micro-environment is imminent. 9 A decellularized scaffold has considerable osteoinductive capacity because it contains several organic components, such as growth factor, fibronectin, heparin sulfate, chondroitin sulfate, and hyaluronic acid. 37 The decellularized method was achieved by cellular washing using a surfactant agent, such as sodium dodecyl sulfate. It functioned as an ionic detergent to eliminate the DNA component in certain tissues, such as bone, by destroying protein binding and initiating cellular membrane lysis, resulting in the destruction of the organic components. 38 The research results demonstrated that dc-FDBB RUNX2 and OSX expression and calcium deposits in the microscopic view were inferior and less calcified when compared with FDBB and DBBM because of extensive organic component destruction. 37 38

DBBM is considered a gold standard in xenografting because it has superior osteoconductive properties with high structural and volumetric stability. DBBM is said to have a low risk of immunorejection due to the heat deproteinization process that eliminates all organic components and only preserves inorganic components, such as calcium and phosphate. 39 The results of this research show that DBBM has superior osteoinductivity, as measured by RUNX2 and OSX expression, when compared with dc-FDBB, but it is still inferior to FDBB. DBBM is considered to be a scaffold without osteoinductive capacity, as it contains no organic components. 40 However, DBBM has free ions of calcium and phosphate that could initiate Notch signaling pathways, further resulting in the upregulation of RUNX2 and OSX expression. 41 FDBB is considered to be an ideal bioactive scaffold that has shown good osteoinductive and osteogenic capacity when compared with dc-FDBB and DBBM. There was no significant difference in osteoinductive markers observed between these scaffolds because all of them activate osteoblastic signaling pathways in a different manner. 13 40 42 The limitation of this study was that observations were only conducted on D6 and D12, while osteogenesis begins on the first day, reaching its peak between day 14 and day 21. 26 35

## Conclusion

FDBB had higher osteoinductive and osteogenic capacity when compared with dc-FDBB and DBBM. Further research is essential to evaluate in vivo bone formation of the FDBB scaffold seeded with mesenchymal stem cells in vivo.
